# Daptomycin versus placebo as an adjunct to beta-lactam therapy in the treatment of *Staphylococcus aureus* bacteremia: study protocol for a randomized controlled trial

**DOI:** 10.1186/s13063-018-2668-6

**Published:** 2018-05-29

**Authors:** Matthew P. Cheng, Alexander Lawandi, Guillaume Butler-Laporte, Katryn Paquette, Todd C. Lee

**Affiliations:** 1 Division of Infectious Diseases, Department of Medicine, McGill University, 1001 Boulevard Décarie E5-1917, Montreal, QC H4A 3J1 Canada; 2 Division of Neonatology, Department of Pediatrics, Sainte-Justine Hospital, Montréal, QC Canada; 3 Clinical Practice Assessment Unit, Department of Medicine, McGill University Health Centre, Montréal, QC Canada

**Keywords:** Daptomycin, *Staphylococcus aureus*, Bacteremia, Beta-lactam

## Abstract

**Background:**

*Staphylococcus aureus* bacteremia is associated with significant morbidity and mortality. To treat this infection, the current standard of care includes intravenous anti-staphylococcal beta-lactam antibiotics and obtaining adequate source control. Combination therapy with an aminoglycoside or rifampin, despite early promise, can no longer be routinely recommended due to an absence of proven benefit and risk of harm. Daptomycin is a rapidly acting bactericidal antibiotic that is approved for the treatment of *Staphylococcus aureus* bacteremia as monotherapy but has not been shown to be superior to the current standard of care. As demonstrated in vitro, the addition of daptomycin to beta-lactam therapy may result in enhanced anti-staphylococcal activity. Our objective is to assess the efficacy and safety of prescribing the combination of daptomycin with cefazolin or cloxacillin for the treatment of methicillin-susceptible *Staphylococcus aureus* (MSSA) bacteremia in adults. We hypothesize that adjunctive therapy with daptomycin will reduce the duration of bacteremia in this population.

**Methods:**

The DASH-RCT trial is a randomized, double blind, placebo-controlled trial designed per the Standard Protocol Items: Recommendation for Interventional Trials (SPIRIT) and Consolidated Standards of Reporting Trials (CONSORT) guidelines. We recruit adults with confirmed MSSA bacteremia, at the McGill University Health Center. Patients are eligible if they are 18 years or older, can receive cefazolin or cloxacillin monotherapy, and are enrolled within 72 h of the first blood culture being drawn. Exclusion criteria include anaphylaxis to study drugs, having polymicrobial bacteremia, anticipated hospital admission for < 5 days, and healthcare team refusal. While receiving standard of care, study patients are randomized to a 5-day course of adjunctive daptomycin or placebo. The trial began in December 2016 and is expected to end in December 2018, after recruiting an estimated 102 patients.

**Discussion:**

The DASH-RCT will compare the use of daptomycin as an adjunct to an anti-staphylococcal beta-lactam versus placebo in the treatment of MSSA bacteremia. We believe that a short course of dual therapy will result in earlier eradication of bacteremia and that subsequent research could evaluate effects on metastatic infection, relapse, and/or mortality. Ongoing issues in the trial include a delay between presentation of infection, enrollment in the trial, and the potential for unrecognized deep foci of infection at diagnosis.

**Trial registration:**

ClinicalTrials.gov, NCT02972983. Registered on 25 November 2016.

Trial protocol: http://individual.utoronto.ca/leet/dash/dashprotocol.pdf

**Electronic supplementary material:**

The online version of this article (10.1186/s13063-018-2668-6) contains supplementary material, which is available to authorized users.

## Background

*Staphylococcus aureus* is a leading cause of bacteremia, with an annual incidence of 38.2 to 45.7 cases per 100,000 people in the USA [1, 2]. Despite effective antibiotics, approximately 20% of patients will die from the initial infection [3–6] and as many as 62% of patients will die within one year [7]. Furthermore, approximately 30% of patients with *S. aureus* bacteremia will develop metastatic infection requiring prolonged antibiotic courses and, in certain cases, surgical intervention. The rates of these complications are increased in those who remain febrile and/or in those in whom bacteremia does not clear within 48 to 72 h [8]. As such, the timely clearance of bacteremia may be an important component in the management of these patients.

The current management of methicillin-susceptible *S. aureus* (MSSA) bloodstream infection consists of adequate source control and anti-staphylococcal beta-lactam (ASBL) antibiotics, notably cloxacillin, nafcillin, or cefazolin [9–12]. The duration of ASBL therapy is determined by the cause of MSSA bacteremia per international clinical practice standards. In short, uncomplicated MSSA bacteremia is treated for 2 weeks, complicated or disseminated infection for at least 4 weeks, and infective endocarditis for a minimum of 6 weeks.

The use of adjuvant gentamicin combined with an ASBL was formerly advocated, as this combination was shown to reduce the duration of MSSA bacteremia [13]. However, due to aminoglycoside-induced nephrotoxicity without a demonstrated reduction in mortality, this combination is no longer recommended in the treatment of *S. aureus* infections outside of prosthetic valve infective endocarditis [14]. Nevertheless, finding another synergistic drug that accelerates bacterial clearance without toxicity could prove a significant finding that could translate into a reduction in subsequent metastatic infection, recurrence, and/or mortality. To this end the role of combination therapy in the treatment of *Staphylococcus aureus* infections remains an area of active research interest. The combination of vancomycin and beta-lactam therapy for the treatment of methicillin-resistant *S*. *aureus* (MRSA) bacteremia has been demonstrated to shorten the duration of bacteremia [15], and there is now an ongoing randomized controlled trial to investigate the potential superiority of daptomycin/vancomycin in combination with beta-lactams in the treatment of MRSA infections with complication-free survival at 90 days as the primary endpoint [16].

Daptomycin is a lipopeptide antibiotic active against both MSSA and MRSA [17, 18]. This antibiotic acts in concert with calcium ions to insert itself into the cell membrane of Gram-positive bacteria, oligomerize, and cause a positive curvature strain on the membrane’s phospholipids. By changing the configuration of the bacterial cellular membrane, daptomycin induces rapid depolarization of the microbe and extravasation of cell contents, and results in cell death [19, 20]. Daptomycin’s synergism with various beta-lactams has been demonstrated for several different Gram-positive cocci, notably *S*. *aureus* and *Enterococcus* species [7, 21–26]. The therapeutically active daptomycin-calcium complex has a net positive charge and must bind to the cell surface in order to exert its therapeutic effect. It has been demonstrated that beta-lactams can reduce a bacterial cell’s surface charge, thereby enhancing the binding of the daptomycin complex to the cell wall and as a result, increasing its bactericidal effect [24]. These interactions have even demonstrated restoration of daptomycin susceptibility to previously non-susceptible *Enterococci* [25]. While much of the potential synergism has been demonstrated in *Enterococci*, there is evidence that this also applies to *S*. *aureus*. For example, it has been shown in vitro that beta-lactams can reduce the net positive charge of the *S*. *aureus* cell membrane, resulting in increased drug binding and a subsequent reduction in the organism’s minimum inhibitory concentration (MIC) to daptomycin [22, 27–29]. Combinations of oxacillin and daptomycin have demonstrated synergistic activity in vitro against MSSA isolates [27] and against MRSA [21, 29]. Thus, treatment with daptomycin and a beta-lactam appears to result in increased bactericidal activity.

Daptomycin is licensed for the treatment of *S*. *aureus* bacteremia including strains that are methicillin-susceptible. However, it is not currently the standard of care due to a lack of direct comparison to ASBLs in the treatment of methicillin-susceptible S. *aureus* bacteremia.

To investigate the potential role of adjuvant daptomycin in the treatment of MSSA infections, we designed the “Daptomycin as Adjunctive therapy for *Staphylococcus aureus* Bacteremia” trial (DASH-RCT). Our objective is to assess if the addition of 5 days of adjunctive daptomycin therapy as compared to ASBL monotherapy will decrease the total duration of MSSA bacteremia in hospitalized men and women 18 years of age or older, with MSSA bacteremia.

## Methods

The DASH-RCT is a randomized, double-blind, placebo-controlled trial enrolling at three McGill University Health Center (MUHC) hospitals: the Montreal General Hospital, the Royal Victoria Hospital, and the Montreal Neurological Institute. The research protocol was submitted to the MUHC Research and Ethics Board who granted approval of the study in November 2016. The trial is funded via internal funds received from the MUHC Association of Physicians. Please see Additional file 1: SPIRIT table in the Appendix for a detailed description of the items included in our clinical trial protocol.

### Study population

Eligible inpatients are aged 18 years or older with documented MSSA bacteremia, who are enrolled within a maximum of 72 h of the culture being drawn and within 24 h of the confirmation of *S*. *aureus*. Patients are excluded if they are moribund due to other illnesses and are expected to die within 5 days of potential recruitment, are clinically appropriate for admission to a critical care unit but are not going to receive critical care due to an advanced directive, or if they are unable to receive either ASBL monotherapy or a combination of ASBL and daptomycin exclusively. The latter precludes individuals with known type I hypersensitivity to the study drugs from participating in the trial and those with polymicrobial infections requiring additional antimicrobial drugs. Patients for whom the use of open-label daptomycin was felt to be indicated by the treating doctors were also excluded.

Our research team is notified of eligible patients via direct page coupled with an automatically generated fax when a new presumptive *S*. *aureus* infection is identified on blood culture. The microbiologic protocols followed to identify *S*. *aureus* and determine methicillin susceptibility are included in the Appendix: Figure 2. After the organism is identified, patients are assessed for eligibility and approached for the study by trained personnel. If patients are unable to consent, the research team approaches their power of attorney for third-party consent.

Upon enrollment, each patient is assigned a unique numeric study identifier that will be used in the subsequent randomization and statistical analysis. Randomization for each unique identifier is performed in advance by permuted block with variable block size. The randomization table is held in confidence by the central research pharmacy, which services all three sites. Investigators and study personnel outside of the research pharmacy do not have access to the randomization table.

### Study protocol

Enrolled patients are randomized to daptomycin and an ASBL or placebo and an ASBL. Daptomycin at a dose of 6 mg/kg body weight is administered intravenously once daily for 5 days in persons with normal renal function (see Tables 1 and 2 for details). This dose was chosen as it is the dose approved by Health Canada for the treatment of *S*. *aureus* bacteremia. In obese patients, the actual body weight is used as in keeping with the monograph. The study drug is dissolved in a 50-mL bag of NaCl 0.9% or in an appropriately sized syringe if there is a minibag shortage. The placebo is NaCl 0.9%, which is provided in the same container and administered intravenously once daily for 5 days in persons with normal renal function; the dosing frequency is adjusted based on the patient’s renal function in the same manner as daptomycin (see Table 2).

**Fig. 1:**
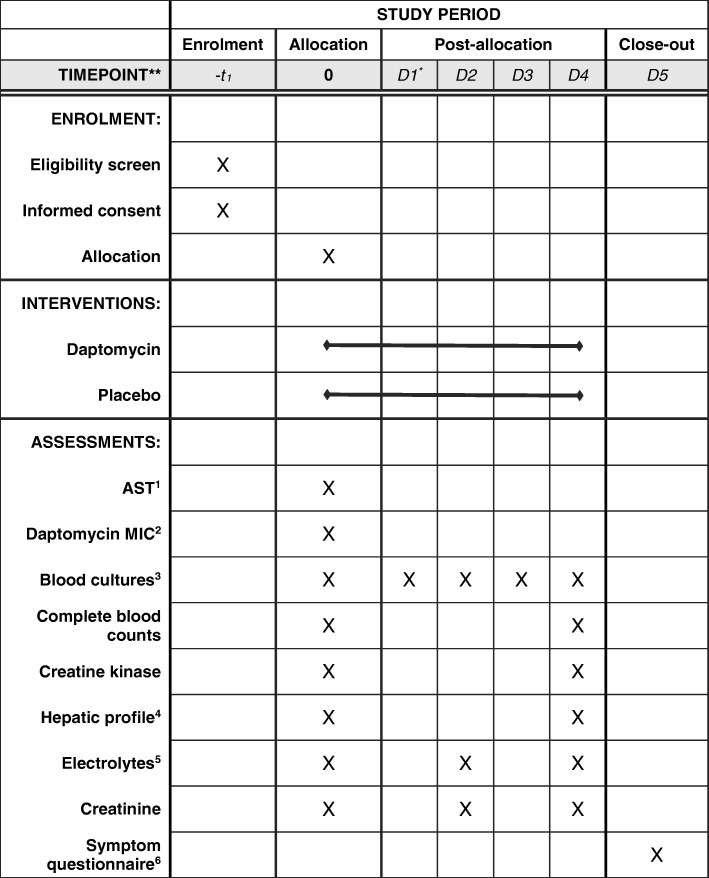
Time and events scheduled for patients enrolled into the DASH-RCT. ^*^Refers to the first day post-allocation. ^1^Routine antimicrobial susceptibility testing is performed on the first methicillin-susceptible *Staphylococcus aureus* isolate of each patient. ^2^The minimal inhibitory concentration (MIC) to daptomycin is performed by Etest® on the first methicillin-susceptible *S. aureus* isolate of each patient. ^3^Two sets of blood cultures are obtained daily for 5 days or until microbiological clearance is documented, whichever is longer. ^4^Hepatic profile includes aspartate aminotransferase, alanine aminotransferase, alkaline phosphatase, gamma-glutamyl transferase, and total bilirubin. ^5^Electrolytes include sodium, potassium, chloride, and bicarbonate. ^6^A symptom questionnaire is administered on day 5 to assess patients for nausea, vomiting, headaches, and myalgia

**Table 1:** Daptomycin dosing in accordance with patient weight

Daptomycin dosing 6 mg/kg body weight	
Weight (kg)	Daptomycin dose (mg)
38–45	250
46–54	300
55–62	350
63–70	400
71–79	450
80–87	500
88–95	550
96–104	600
105–112	650
113–120	700
121–129	750
130–137	800
138–145	850
More than 145	900

**Table 2:** Daptomycin dosing interval per patient renal function

Renal function	Dosing interval
Creatinine clearance > 30 mL/minute	Q24H × 5 doses
Creatinine clearance < 30 mL/minute	Q48H × 3 doses
Continuous renal replacement therapy	Q24H × 5 doses
Peritoneal cialysis	Q48H × 3 doses
Intermittent hemodialysis	Qdialysis × 3 doses

The MUHC research pharmacy monitors the creatinine clearance of enrolled patients and contacts the research team to suggest dosing modifications as needed, regardless of drug allocation. The study drug is delivered to the patient’s admitting unit in the form of a sealed container with the patient’s name and unit number and administration instructions.

The choice of ASBL, either cloxacillin or cefazolin, is at the treating team’s discretion. Further management of the infection is also at the treating team’s discretion. The study protocol does not prescribe the timing of removal of intravascular devices, performance of echocardiography, ancillary investigations aside from the bloodwork mentioned below, or consultation with an infectious diseases specialist. Statin therapy may be continued.

The study mandates that regular blood samples be obtained to document clearance of the infection and monitor for drug adverse effects (see Fig. 1). Two sets of aerobic and anaerobic blood cultures are obtained daily for 5 days or until microbiological clearance is documented. As per our routine hospital practice, the first blood culture set contains both an aerobic and an anaerobic bottle, whereas the second set contains only an aerobic bottle. Complete blood counts, creatine kinase, aspartate aminotransferase, alanine aminotransferase, alkaline phosphatase, gamma-glutamyl transferase, and total and direct bilirubin are drawn on days 1 and 5. Electrolytes and creatinine are measured at a minimum on days 1, 3, and 5. In addition, each patient’s first MSSA isolate undergoes full antimicrobial susceptibility testing, and testing of the MIC to daptomycin using the Daptomycin ETest (BioMérieux, France).

The patients, their clinicians, the investigators assessing the primary outcome, and those performing the statistical analysis are blinded to the treatment allocation. Unblinding can be requested by the treating physician in the event of a serious adverse event suspected to be due to the study drug. The research team is notified that the patient has been unblinded and excluded from receiving further study drug. However, the patient’s allocation arm remains unknown to the investigators. Such patients are maintained in the intention-to-treat analysis.

The participants’ data are collected and managed using REDCap electronic data capture tools hosted at the McGill University Health Center [30]. Data are anonymized and de-identified to ensure patient confidentiality.

### Outcomes

The primary outcome is to assess if the addition of 5 days of daptomycin adjunctive to an ASBL compared to ASBL monotherapy will decrease the duration of MSSA bacteremia in hospitalized men and women 18 years of age or older. The total duration of bacteremia will be determined based on microbiology laboratory reports. The duration will be defined in days, where day 1 is the day on which the first positive blood culture was drawn and the final day is the last day that yielded a positive blood culture.

Secondary outcomes are as follows:

1. All cause 30-day mortality will be obtained from hospital records and centralized provincial health care databases.
2. Relapsed bacteremia is defined as a blood culture specimen positive for MSSA obtained ≥ 48 h and  ≤ 90 days after obtaining a negative blood culture (negative after 5 days of incubation). Results will be obtained from the electronic microbiology laboratory records.
3. Clinically relevant embolic or metastatic MSSA disease will be diagnosed within 90 days of the first positive blood culture by the treating team and defined as the presence of bone abscess or osteomyelitis on computed tomography (CT), magnetic resonance imaging (MRI), or bone and gallium scan; renal emboli or ilio-psoas, splenic, and other occult abscesses on ultrasound, CT or MRI; septic brain emboli on CT of the head or MRI of the brain; septic emboli to the lung on chest x-ray or CT; or infective endocarditis on echocardiography.
4. The patient safety outcome will be a combined endpoint of nephrotoxicity, as defined by the Kidney Disease: Improving Global Outcomes (KDIGO) guidelines [31]; hepatotoxicity, as defined by an increase in liver enzymes three times the upper limit of normal; and rhabdomyolysis, as defined by an increase in creatine kinase three times the upper limit of normal, occurring within the first 5 days of therapy. A questionnaire administered on day 5 post-allocation is used to assess patients for nausea, vomiting, headaches, and myalgia.

### Statistical analyses

Sensitivity analysis was performed to determine the sample size required to achieve 80% power. Assuming that the duration of bacteremia was normally, log-normally, or Poisson distributed, with a mean duration of bacteremia of 3 days, and a standard deviation of 2 days (3 for Poisson), a sample size of approximately 102 participants would be required to detect a 1-day difference in duration of bacteremia at a significance level of 5%. If the mean duration of bacteremia is 2 days, we would have 70% power for our primary outcome assuming the duration of bacteremia was normally or log-normally distributed. Assumptions were adapted from a previously published study [15] and derived from our center’s local epidemiology.

Participants will be analyzed according to their treatment allocation in an intention-to-treat analysis. A modified intention-to-treat analysis will exclude participants whose blood cultures were already negative at the time of their first study drug dose. Participants who have received at least 3 days of therapy according to their treatment allocation will also be analyzed in a per-protocol fashion as part of the secondary analyses.

Primary outcome: the duration of MSSA bacteremia will be evaluated via time-to-event analysis using Kaplan-Meier curves reporting the median time to clearance. Comparisons will be made using the Tarone-Ware log rank test with significant α < 0.05.

Secondary outcomes are as follows:

1. The proportion of patients who are clear of MSSA bacteremia at days 3, 5, and 7.
2. Relapsed bacteremia will be a binary outcome; proportions will be reported, and will be compared using Fisher’s exact test.
3. Metastatic MSSA disease will be a binary outcome; proportions will be reported, and will be compared using Fisher’s exact test.
4. The patient safety outcome will be binary; proportions will be reported, compared using Fisher’s exact test, then we will adjust for confounders using exact logistic regression.
5. Analysis of effect modification of the primary outcome using Cox regression models: we will analyze patient comorbidities, including immunosuppressive conditions, the presence of endocarditis, source control difficulties, metastatic MSSA infection, and an infectious diseases consultation.
6. Accounting for confounders: imbalances in potential confounders between study arms will be evaluated for their influence on the relationship between the intervention and the time to clearance of bacteremia using Cox regression models. Exact logistic regression will be used to adjust for confounders of the secondary outcomes (numbers 1 through 4).
7. Per-protocol analysis of the time to clearance of bacteremia between the study groups: time-to-event analysis using Kaplan-Meier curves, comparisons using the Tarone-Ware log rank test.

An interim analysis is planned upon enrollment of 50 patients who continue to have bacteremia at the time of enrollment. This analysis will be reviewed by an independent Safety Monitoring Board comprising two physicians and one statistician who are not appointed to the division of Infectious Diseases and are not involved in the study. They have the authority to stop the study if there is significant evidence of benefit or harm (primary outcome, primary safety outcome) based on the O’Brien-Fleming alpha-spending approach [32].

All statistical analyses will be performed using statistical software including STATA 15.0 (STATA Corp, College Station, TX, USA), SAS 9.4 (SAS Institute Inc., Cary, NC, USA), and/or R (R Foundation for Statistical Computing, Vienna, Austria).

## Discussion

The objective of the DASH-RCT is to assess whether patients receiving an ASBL and daptomycin are at reduced risk of prolonged bacteremia. We hope that an improvement in this endpoint might be associated with a reduction in metastatic infections, recurrence, and mortality. Our goal is to assess whether daptomycin is a good candidate to be studied in a larger study powered for those outcomes. Ultimately, we would like to determine whether the standard of care for MSSA bacteremia should become combination therapy with an ASBL and daptomycin.

There are several limitations to the DASH-RCT trial. First, the identification of MSSA in blood cultures and the subsequent enrollment of the patient in the trial is delayed by the need to identify the microorganism in the laboratory. As a result, patients may be exposed to a variety of open-label broad-spectrum antibiotics prior to trial enrollment for up to 72 h. While we believe there will not be bias in these between the arms of the study due to randomization, there is a possibility of such imbalance, which will need to be considered. We have put into place techniques (see Appendix) for early identification of MSSA from positive blood cultures and enroll on evenings and weekends to minimize delays. Unfortunately, these early identification techniques could lead to early misidentification of MSSA in approximately 3% of patients (based on unpublished but extensive local verification studies) and those patients would need to be excluded. However, using our modified tube coagulase test (Appendix), rapid MRSA testing (Appendix), and accounting for known MRSA colonization status [33] allows us to recruit patients into the study as close to presentation as possible, while maintaining specificity. Second, as the organism may have already disseminated at the time of species identification, the ability to evaluate whether daptomycin can limit metastatic spread may be diminished. We chose to assess patients for clinically relevant embolic or metastatic MSSA disease as evaluated by the treating team, which has been previously validated as a clinical outcome in *S*. *aureus* trials [34]. While we record clinically significant metastatic events at enrollment and follow up, for cost and diagnostic stewardship reasons we will not perform extensive imaging at the time of randomization nor systematically search for embolic disease during the trial, which is in keeping with other large randomized controlled trials involving *S*. *aureus* [17, 35]. As we do not anticipate differential allocation of patients with early metastatic disease to a specific arm, a difference in clinically relevant events between groups would be suggestive (but not definitive) of a drug effect. This secondary endpoint should thus remain informative despite the absence of additional diagnostic procedures performed throughout the study.

To mitigate the previously mentioned limitations, we could have enrolled all patients with presumed staphylococcal infection per the presence of Gram-positive cocci in clusters on Gram stain of positive blood cultures. Rapid enrollment would have reduced the time between presentation and initiation of the study drugs at the cost of increased post-randomization exclusions. Indeed, more patients would have been recruited with MRSA blood stream infections or blood culture contamination due to coagulase-negative *Staphylococcus*. Advances in earlier diagnosis using a molecular microbiologic technique could provide the opportunity for earlier enrollment without enrolling patients with non-relevant Gram-positive cocci.

Second, many patients enrolled in the trial will be expected to be clear of bacteremia by the time of enrollment, as only approximately 30–40% of Staphylococcal bacteremia persists. It is very difficult to predict in advance who these patients will be and the study sample size has been chosen to allow for this. Due to the lag in identification of bacteremia, it is impossible to avoid the enrollment of patients who may have already be clear of bacteremia at enrollment. Nevertheless, this limitation will apply to both the study group and the placebo group and therefore the role of adjunctive daptomycin in mitigating complications of the bacteremia will still be of interest. Moreover, we have also pre-planned a modified intention-to-treat analysis to look only at those who still have bacteremia when they start the study drug.

Despite the aforementioned limitations, we believe that the DASH-RCT is founded in rigorous methodology and examines a biologically plausible hypothesis. Our study may demonstrate that daptomycin in combination with an ASBL results in faster clearance of MSSA bacteremia than the current standard of care. Prescribing a short course of daptomycin at the Health Canada and U.S. Food and Drug Administration approved dose will limit potential dose-dependent toxicities. Benefits similar to combination therapy with aminoglycosides may then be demonstrated, but with reduced rates of adverse events. If our hypotheses hold true, further trials to evaluate daptomycin as an adjunctive therapy in the treatment of MSSA-associated diseases will be warranted. However, given that the mortality associated with MSSA bacteremia is approximately 20%, a significantly larger randomized controlled trial would be required to demonstrate a survival benefit of using combination therapy.

## Trial status

The DASH-RCT began enrolling patients on 1 December 2016. The trial is ongoing. Patient recruitment and follow up is expected to be completed by 31 December 2018.

### Additional file

Additional file 1: SPIRIT 2013 Checklist: Recommended items to address in a clinical trial protocol and related documents*. (DOC 120 kb)

## Figures and Tables

**Fig. 2:**
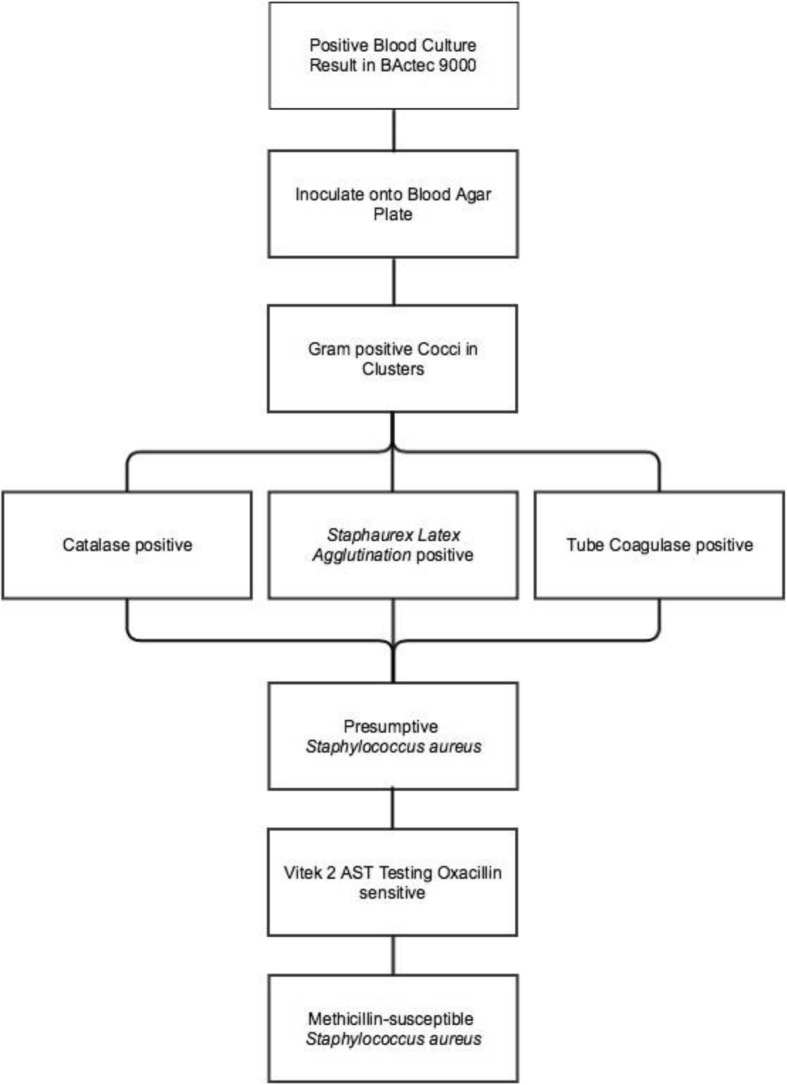
Identification algorithm utilized for *methicillin-susceptible Staphylococcus aureus*

## Appendix

Presumptive identification of *methicillin-susceptible Staphylococcus aureus*

1. Inoculated blood culture bottles (Bactec, Becton, Dickinson and Company, New Jersey, USA) are incubated in the Bactec 9000 blood culture instrumentation system as per manufacturer instructions until identified as positive.
2. Positive bottle are removed from the incubator and plated onto blood agar.
3. Colonies are gram-stained At 24 h.
4. Isolates that are beta hemolytic, gram-positive cocci in clusters are then tested for the presence of catalase and coagulase using conventional biochemical analysis.
5. These isolates are screened for clumping factor using the *Staphaurex Latex Agglutination Assay* (ThermoFisher Scientific, MA, USA).
6. A tube coagulase is also performed on these and the results read at 4 h.
7. Isolates are identified as presumptive *S*. *aureus* when they are found to be beta-hemolytic, gram-positive cocci in clusters, which are catalase-positive, latex agglutination-positive, and tube coagulase-positive.
8. Antimicrobial susceptibility and oxacillin screening is performed on the Vitek 2 system using their Antimicrobial Susceptibility Cards (AST) (BioMerieux, Marcy-l’Étoile, France). *Methicillin-susceptible S*. *aureus* is identified when isolates are reported as sensitive to oxacillin using this system.

## Abbreviations

ASBL: Anti-staphylococcal beta-lactam
BSI: Bloodstream infections
CT: Computed tomography
MIC: Minimum inhibitory concentration
MRI: Magnetic resonance imaging
MRSA: Methicillin-resistant *Staphylococcus aureus*
MSSA: Methicillin-susceptible *Staphylococcus aureus*
MUHC: McGill University Health Center
